# Validated Stability indicating Spectrophotometric Method for the Determination of Acetazolamide in Dosage Forms through Complex Formation with Palladium (II)

**Published:** 2010-06

**Authors:** M. I. Walash, A. El-Brashy, N. El-Enany, M. E. K. Wahba

**Affiliations:** *Department of Analytical Chemistry, Faculty of Pharmacy, Mansoura University, Mansoura, Egypt*

**Keywords:** spectrophotometry, Palladium (II), complexation, acetazolamide

## Abstract

A simple and sensitive spectrophotometric method was developed for the determination of acetazolamide (ACM) in pure form and pharmaceutical preparations. The proposed method is based on the complex formation of acetazolamide with Palladium (II) chloride in acetate buffer pH5.4 and measuring the absorbance at 308 nm. The absorbance- concentration plot was rectilinear over the concentration range of 5-70 μg/ml with a minimum detection limit (LOD) of 0.98 μg/ml, limit of quantification (LOQ) of 2.96 μg/ml, and a molar absorptivity ζ=2.7 × 10^3^ L/mol.cm. The factors affecting the absorbance of the formed complex were carefully studied and optimized. The composition of the complex as well as its stability constant was also investigated. The proposed method was applied for the determination of acetazolamide in its tablets and the results obtained were favorably compared with those obtained using the official method. A proposal of the reaction pathway was postulated.

## INTRODUCTION

**Figure 1:**
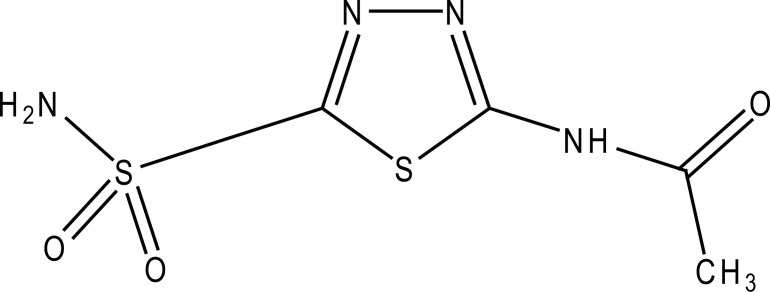
Structural formula of acetazolamide.

Acetazolamide (ACM) is chemically named according to the IUPAC system as 5-acetamido-1, 3, 4-thiadiazole-2-sulphonamide; N-(5-sulphamoyl-1, 3, 4, thiadiazole-2-yl) acetamide (Fig. 1). It acts on the central nervous system to retard the abnormal, paroxysmal, and excessive discharge from CNS neurons (1). Various methods were reported for its determination either *per se* or in pharmaceutical preparations and biological fluids including: spectrophotometry (2-6), HNMR (4), electrochemical methods (7, 8), and HPLC (9-27). The British Pharmacopoeia (BP) (28) recommended potentiometric determination of ACM in dimethylformamide using ethanolic solution of sodium hydroxide as a titrant, while the United States Pharmacopoeia (USP) method (29) recommended a spectrophotometric measurement of the acidic solution of the pharmaceutical preparation at 265 nm. Since the hydrolysis product 5-amino-1, 3, 4 - thiadiazole-2-sulfonamide also absorbs light at the same wavelength (265 nm); the USP method therefore cannot be considered as a stability indicating assay (29). A good guide to the work published for this compound up to 1993 is found in the monograph written by Parasrampuria on its Analytical Profiles of Drug Substances and Excipients (30).

The use of Palladium (II) chloride as a complexing agent for the determination of many compounds of pharmaceutical interest is fairly wide. The complexes of palladium (II) ions bear the advantage of being water-soluble, and hence do not necessitate any extraction procedures. Several compounds of pharmaceutical interest were determined throught this approach such as: captopril (31), gliclazide (32), some thiazole cephalosporins (33), norfloxacin and ciprofloxacin (34), phenothiazines (35-37), timonacic acid (38), and thioctic acid (39).

The molecular structure of ACM is characterized by the presence of sulfur atom which is susceptible to complexation with palladium (II) and this initiated the present study. Up to the present time, nothing has been published concerning the complex formation between ACM and Pd (II).

Since the proposed method proved to be a stability indicating one, it could be a useful guide to indicate the presence of acetazolamide in its stable intact form keeping in mind that the hydrolyzed form of the drug has a little if any pharmacological activity.

## EXPERIMENTAL PROCEDURES

### Apparatus

The spectrophotometric measurements were established using Shimadzu UV-visible 1601 recording spectrophotometer (P/N 206-67001). Recording range, 0-1.0; wavelength, 308 nm.

### Materials and reagents

All reagents and solvents were of Analytical Reagent grade. Acetazolamide (ACM) was kindly provided by Chemical Industries Development (CID) pharmaceutical company, Cairo, Egypt. Its purity was checked according to BP (28) and was found to be 98.8%. Cidamex tablets (Batch# 107101), labeled to contain 250 mg of ACM per tablet (CID pharmaceutical company, Cairo, Egypt), and were obtained from commercial sources in the local market. Palladium (II) chloride (Sigma, Milwaukee, WI, USA) was prepared as 5 × 10^-3^ M solution by dissolving about 88.4 mg of PdCl_2_ in 1 ml hydrochloric acid, with the aid of heat, followed by the addition of boiled water and diluting to 100 ml with distilled water. This solution is stable for two weeks when kept in the refrigerator. Acetate buffer (pH3.6-5.6) (28) was prepared by mixing 0.2 M acetic acid and 0.2 M sodium acetate, the pH has to be checked periodically. Methanol and hydrochloric acid (Merck, Darmstadt, Germany). Potassium chloride, 2 M aqueous solution (BDH, UK), freshly prepared was used.

### Standard solutions

A stock solution was prepared by dissolving 100.0 mg of ACM in 100 ml of methanol and was further diluted with the same solvent as appropriate. The standard solutions were stable for 7 days when kept in refrigerator.

### Construction of the calibration curve

Aliquot volumes of ACM standard solution covering the working concentration range cited in Table 1 were transferred into a series of 10 ml volumetric flasks. 2.5 ± 0.5 ml of acetate buffer pH5.4, followed by 1 ± 0.2 ml of PdCl_2_ (5 × 10^-3^ M) solution, and 2 ml of 2 M KCl solution were added, and the volume was completed to the mark with distilled water, and mixed well. The absorbance of the solution was measured at 308 nm against similarly prepared blank solution. Base line correction was carried out to delete any absorbance reading of the blank. The measured absorbance values were plotted against the final concentration of the drug (μg/ml) to get the calibration curve. Alternatively, the corresponding regression equation was derived.

### Procedure for tablets

Twenty tablets were weighed and pulverized. A weighed quantity of the powder equivalent to 20.0 mg of ACM was transferred into a small conical flask, extracted three successive times each with 30 ml of methanol. The extract was filtered into 100 ml volumetric flask. The conical flask was washed with few mls of methanol. The washings were passed into the same volumetric flask and completed to the mark with the same solvent. Aliquots covering the final concentration range over 10-60 μg/ml were transferred into 10 ml volumetric flasks. The procedures described under “Construction of calibration curve” were followed. The nominal content of the tablets was determined either from the calibration curve or using the corresponding regression equation.

### Procedure for preparation of the alkaline degradation product

For the kinetic study, aliquot volumes of ACM standard stock solution were transferred to a 25 ml volumetric flask to obtain a final concentration of 400 μg/ml, completed to the volume with 0.5 M sodium hydroxide, and the solution was left in a boiling water bath. Aliquot volumes of the alkaline solution were transferred to a series of 10 ml volumetric flasks at a fixed time interval (10 minutes), neutralized with 0.5 M hydrochloric acid, and the procedures described under “Construction of the calibration curve” were followed. The absorbance of the resulting hydrolyzed solutions after complexation with Pd (II) was recorded at 308 nm, log a/a-x versus time (minutes) was plotted to get the reaction rate constant and the half life time. Complete degradation was attained by following the same procedure but using 2 M sodium hydroxide, boiling for 2 hours, and neutralizing with 2 M hydrochloric acid.

## RESULTS AND DISCUSSION

**Figure 2:**
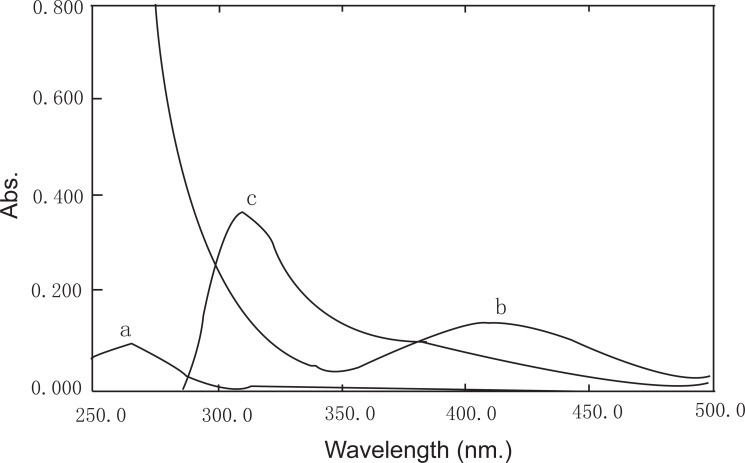
Absorption spectra of: a, Acetazolamide only (30 μg/ml) in acetate buffer pH5.4; b, PdCl_2_ (5 × 10^-3^ M) only; c, The formed complex of ACM (30 μg/ml) with Pd (II) at pH5.4.

Transition elements were found to form stable complexes with many ligands containing heteroatoms. There is preference to amines, halogens, cyanides, tertiary phosphrines and sulfides. Palladium (II), as one of the transition elements, was reported to form complexes of square or 5-coordinate shape (40). The absorption spectrum of the complex was recorded over the range of 250-500 nm and showed a maximum absorbance at 308 nm which was therefore used for the analytical determination (Fig. 2). The formed complex was found to be stable for more than 90 minutes. The proposed method proved to be 53% more sensitive than previous report (20).

### Optimization of the experimental parameters

The spectrophotometric properties of the formed complex as well as the different experimental parameters affecting its development and stability were carefully studied and optimized. Such factors were changed individually while the others were kept constant. These factors include: different types of buffers, pH and volume of the buffer, concentration and volume of palladium (II), temperature, and the effect of different surfactants and different sensitizers.

### Effect of pH and buffer solutions

**Figure 3:**
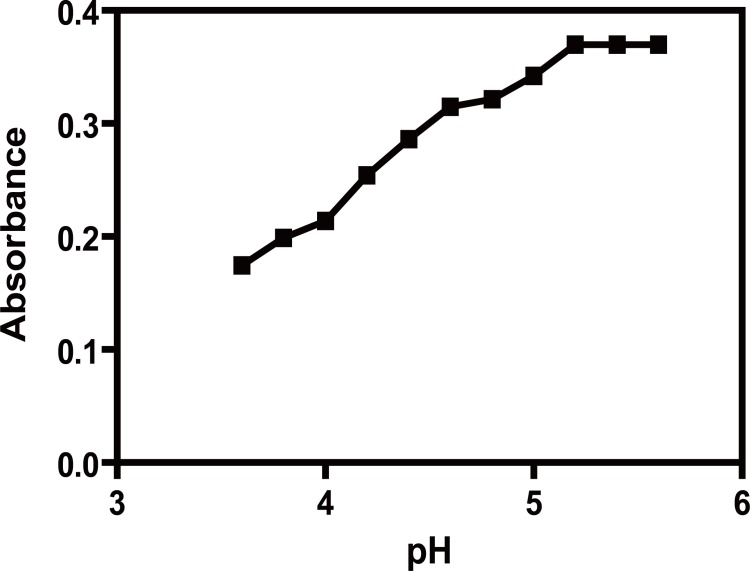
Effect of pH on the absorbance of ACM-Pd (II) complex, (ACM=30 μg/ml).

The study was performed using 30 μg/ml of ACM and 5 × 10^-4^ M Pd (II) final concentration. The influence of pH of the acetate buffer on the absorbance value of the formed complex was investigated over the pH range 3.6-5.6. Maximum and constant absorbance value was achieved at pH(5.2-5.6) (Fig. 3). Therefore, acetate buffer pH5.4 ± 0.2 was used as the optimum pH throughout the study.

Using different buffers such as phosphate buffer or Britton Robinson buffer of pH 5.4 gave the same results; however, acetate buffer was chosen throughout the study because of absence of possible interference produced by other buffers.

### Effect of buffer volume

**Figure 4:**
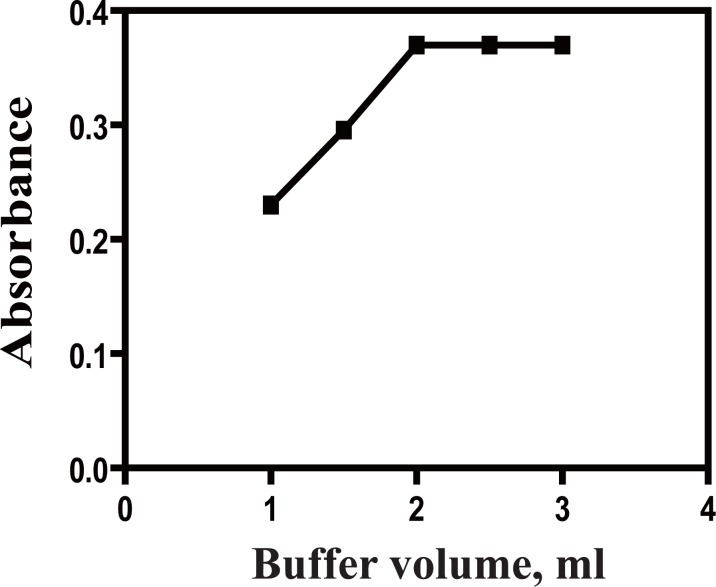
Effect of volume of acetate buffer pH5.4 on the absorbance of ACM-Pd (II) complex, (ACM=30 μg/ml).

The effect of volume of acetate buffer of pH5.4 on the absorbance value of the formed complex was also studied keeping the concentration of the drug and Pd (II) constant. It was found that increasing the volume of acetate buffer (pH5.4) resulted in a subsequent increase in the absorbance value of the complex up to 2.0 ml, after which it remained constant. Thus 2.5 ± 0.5 ml of acetate buffer of pH5.4 ± 0.2 was chosen as the optimum volume throughout this approach (Fig. 4).

### Effect of Palladium (II) concentration

**Figure 5:**
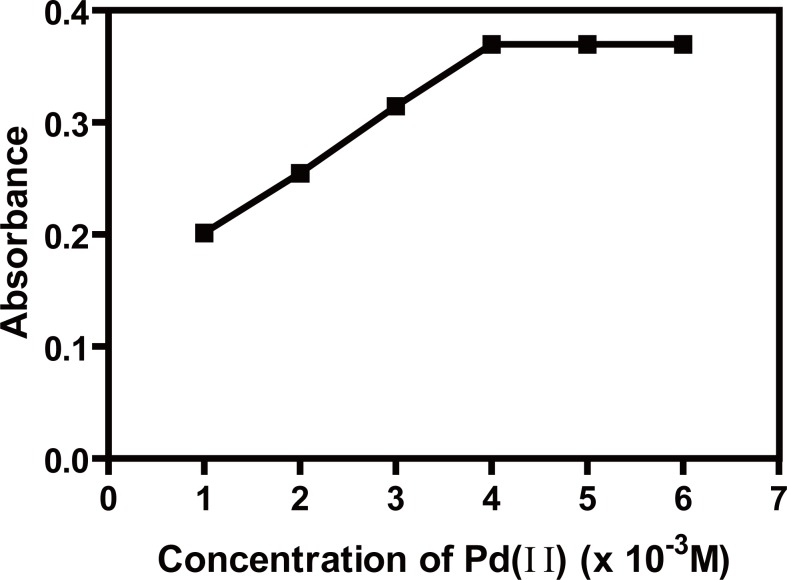
Effect of concentration of Pd (II) (× 10^-3^M), on the absorbance of ACM-Pd (II) complex, (ACM=30 μg/ml) at pH5.4.

The effect of Pd (II) concentration on the absorbance of the complex was investigated, keeping all the variables constant, it was found that increasing the concentration of Pd (II) (1.0 × 10^-3^ M; 1.0 ml) resulted in a gradual increase in the absorbance of the complex up to (4.0 × 10^-3^ M) after which, it remained constant up to (6.0 × 10^-3^ M); therefore; (5.0 ± 1.0 × 10^-3^ M) was used throughout the study (Fig. 5).

### Effect of palladium (II) volume

**Figure 6:**
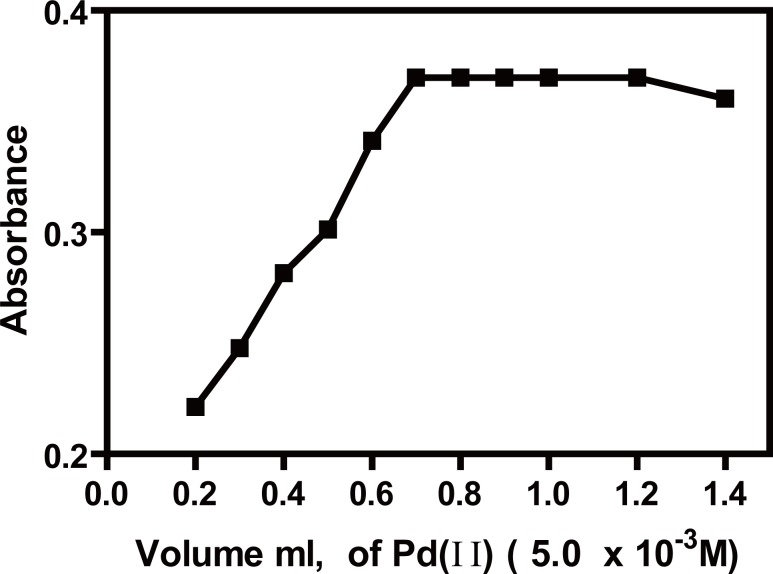
Effect of volume of Pd (II) (5.0 × 10^-3^ M), on the absorbance of ACM-Pd (II) complex, (ACM=30 μg/ml) at pH5.4.

Keeping all the variables constant, it was found that increasing the volume of Pd (II) (5.0 × 10^-3^ M) resulted in a gradual increase in the absorbance value of the complex up to 0.7 ml, after which it remained constant up to 1.25 ml, therefore 1.0 ± 0.2 ml of (5.0 × 10^-3^ M) was chosen for the study (Fig. 6).

The effect of temperature on the absorbance of the formed complex was studied; the absorbance remained constant when the temperature was raised up to 60°C; the complex was formed instantaneously and remained stable for more than one hour at room temperature.

### Effect of addition order

The effect of addition order on the absorbance intensity of the system was studied. The results show that the addition order of ACM- acetate buffer -Pd (II) was the best.

### Effect of different surfactants and sensitizers

The effect of surfactants (at concentrations 2.5, 7.5, 15 μg/ml) on the absorbance value of the formed complex was investigated using different types, such as sodium lauryl sulphate (anionic type), gelatin, and methyl cellulose (non ionic types). Hopefully the surfactant may enhance the absorbance readings of the complex, but unfortunately, it was found that all the studied surfactants had no significant effect on the absorbance of the formed complex. Therefore, for a simple procedure, there is no need to use surfactants. Similarly different sensitizers were tested such as quinine, fluorescein and rhodamine-B. Addition of sensitizers to the reaction mixture was found to enhance the absorbance but with the lack of reproducibility. Therefore, the study was carried out without the addition of sensitizers.

### Analytical performance and application

**Figure 7:**
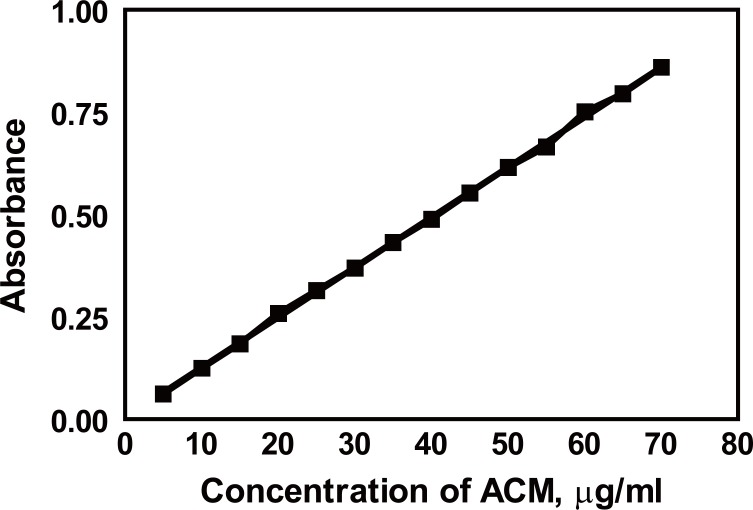
Calibration graph of ACM using the proposed method.

The absorbance –concentration plot was found to be linear over the range of 5.0-70 μg/ml (Fig. 7). linear regression analysis of the data gave the following equation:

$A=0.012C+4.0\times 10^{-3}\;\left(r=0.9997\right)$

where A is the absorbance in 1-cm cell, C is the concentration of the drug in μg/ml, and r is the correlation coefficient.

The limit of quantification (LOQ) was calculated by establishing the lowest concentration that can be measured according to ICH Q2B recommendations (41), and it was found to be 2.96 μg/ml.

LOQ was calculated according to the following equation (41):

LOQ=10σ/S

where σ is the standard deviation of the intercept of the regression line, S is slope of the calibration curve.

The limit of detection (LOD) was calculated according to the ICH recommendations (41) and it was found to be 0.98 μg/ml.

LOD was calculated according to the following equation (41):

LOD=3.3 σ/S

The proposed method was evaluated by studying the accuracy as percent relative error and precision as percent relative standard deviation (RSD %); the results are abridged in Table 1.

**Table 1:** Performance data of the proposed method

Parameter	Value

Concentration range(μg/ml)	5.0-70
Molar absorptivity € (L mol^-1^.cm^-1^)	2.7 × 10^3^
LOD (μg/ml)	0.98
LOQ (μg/ml)	2.96
Correlation coefficient (r)	0.9997
Slope	1.22 × 10^-2^
Intercept	1.19 × 10^-2^
S_y/x_ (Standard deviation of the residuals).	7.3 × 10^-3^
S_a_ (Standard deviation of the intercept of the regression line).	3.6 × 10^-3^
S_b_ (Standard deviation of the slope of the regression line).	9.6 × 10^-5^
% Error(RSD%/ √n).	0.07
% RSD	0.26

### Validation of the method

The method was tested for linearity, selectivity, accuracy and precision. By using the above spectrophotometric procedure, a linear regression equation was obtained. The regression plot showed that there was a linear dependence of the absorbance value on the concentration of the drug over the range cited in Table 1. The validity of the proposed method was evaluated by statistical analysis of the regression data regarding the standard deviation of the residual (S_y/x_), the standard deviation of the intercept (S_a_), and standard deviation of the slope (S_b_) (42). The results are shown in Table 1. The small values of the figures point to the low scattering of the points around the calibration graph and high precision of the proposed method.

### Accuracy

The accuracy of the proposed method was evaluated by analyzing standard solutions of ACM. The results obtained by the proposed method were favorably compared with those obtained by the USP method (29). The latter involved a spectrophotometric measurement of ACM in 0.1 N hydrochloric acid at 265 nm.

Statistical analysis (42) of the results obtained by the proposed and official methods using student’s t-test and variance ratio F-test, showing no significant difference between the performance of the two methods regarding the accuracy and precision, respectively (Table 2).

**Table 2:** Application of the proposed and official method to the determination of ACM in pure form

Parameter	Amount taken (μg/ml)	Amount found (μg/ml)	% Found	Official method (29)

	10	10.12	101.20	100.99
	20	19.98	99.90	101.05
	30	29.86	99.51	99.95
	40	40.08	100.20	100.66 ± 0.43
	50	50.05	100.11	
X ± SD			100.18 ± 0.63	
Student’s t test			0.71 (2.78)	
Variance ratio F test			2.15(6.94)	

Figures between parentheses are the tabulated t and F values, respectively, at *p*=0.05 (42).

### Precision

**Repeatability.** The repeatability was evaluated through the replicate analysis of ACM samples, pure drug or tablets. The percentage recoveries based on the average of four separate determinations were 100.4 ± 0.77 and 99.8 ± 0.32 for pure form and tablets respectively, thus indicating the high precision of the method (Table 3).

**Table 3:** Validation of the proposed method for the determination of acetazolamide in pure form and in tablets

Sample	% Found Repeatability (30.0 μg/ml)	% Found Intermediate precision (40.0 μg/ml)

Acetazolamide (pure form)	99.90	100.51
	100.91	99.71
	101.21	100.90
	99.62	100.32
Mean found %	100.40	100.40
±SD	0.77	0.50
RSD %	0.77	0.50
Cidamex^®^ tablets (acetazolamide 250 mg/tablet)	99.50	98.92
	99.90	99.42
	100.21	98.71
	99.62	99.10
Mean found %	99.80	99.10
± SD	0.32	0.29
RSD %	0.32	0.29

**Intermediate precision.** It was performed through replicate analysis of ACM samples, pure drug or tablets on four successive days. The percentage recoveries based on the average of four separate determinations were 100.4 ± 0.50 and 99.1 ± 0.29 for pure form and tablets respectively. The results are shown in Table 3.

**Robustness of the method.** The robustness of the method adopted was demonstrated by the consistency of the absorbance values with the deliberately minor changes in the experimental parameters such as pH5.4 ± 0.2 produceed a constant absorbance value of 0.37 at constant ACM concentration (30 μg/ml); changing the volume of acetate buffer 2.5 ± 0.5 ml (pH5.4), and volume of Pd (II); 1.0 ± 0.2 ml (5.0 × 10^-3^M). These minor changes that may take place during the experimental operation didn’t greatly affect the absorbance value of the reaction product.

**Pharmaceutical applications.** The proposed method was further applied to the determination of ACM in its tablets.

**Selectivity.** Common tablet excipients such as talc, lactose, starch, avisil, and magnesium stearate did not interfere with the assay. The results are abridged in Table 4.

**Table 4:** Application of the proposed and official method for the determination of ACM in its tablets

Pharmaceutical preparation	Amount taken (μg/ml)	Amount found (μg/ml)	% Found	Official method (29)

Cidamex^®^ tablet (Acetazolamide 250 mg/tablet)	10	10.01	100.10	100.23
	20	19.97	99.85	99.65
	30	30.04	100.13	101.54
	40	39.96	99.90	
	50	49.67	99.34	
	60	60.14	100.23	
X ± SD			99.86 ± 0.32	100.47 ± 0.35
Student’s t test			0.35 (2.31)	
Variance ratio F test			1.19 (5.79)	

Figures between parentheses are the tabulated t and F values, respectively, at *p*=0.05 (42). Cidamex^®^ is a product of Chemical Industries Development (CID) pharmaceutical company, Cairo, Egypt.

### Accuracy

The results of the proposed method were statistically compared with those obtained using the official method. Statistical analysis of the results, using student’s t-test and variance ratio F- test, revealed no significant difference between the performance of the proposed and official methods (29) regarding the accuracy and precision respectively (Table 4).

The formation constant of the reaction product was calculated according to the following equation:

$K_{f}=\frac{A/A_{m}}{\left[{\left(1-A/A_{m}\right)}^{n+1}\right]c^{n}n^{n}}$

where A and A_m_ are the observed maximum absorbance and the absorbance obtained from the extrapolation of the two lines obtained from Job’s continuous variation method, respectively; n is the mole fraction of the reagent (the ratio is 2:1 for ACM: Pd (II) respectively) therefore, n=0.3; C is the molar concentration of the drug used in Job’s continuous variation method.

Using the above equation K_f_ was found to be 0.919 × 10^2^.

Also, the Gibbs free energy changes (ΔG) of the reaction were calculated according to the following equation (43):

ΔG= - 2.303 RT log K_f_

where R is gas constant= 8.3 joule.degree^-1^.mole^-1^; T is absolute temperature= °C + 273.

Using the above equation ΔG was found to be -1.1 × 10^4^ Joule/Mole.

The negative value of ΔG indicates that the reaction is spontaneous.

### Stability indication of the method

**Figure 8:**
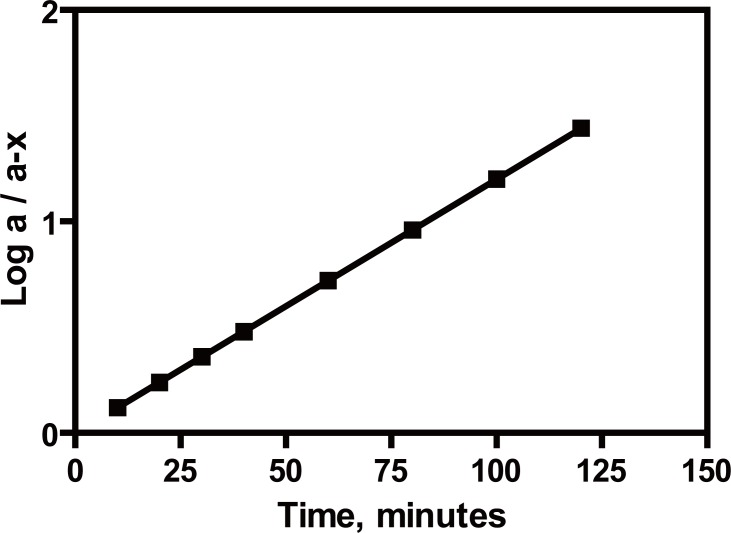
Semi log plot of acetazolamide (30 μg/ml) *versus* different heating times with 0.5 M sodium hydroxide at 100°C.

The proposed method is based mainly on the complex formation between Pd (II) and sulfur atom, imide and sulfonamide groups of the thiadiazole ring of ACM, therefore, upon photoinduced alkaline degradation of the drug, the imide group is decomposed into a primary amine and acetic acid, hence, the complex will no further be formed. Complete degradation of the drug was revealed by the disappearance of the absorption peak of the complex after boiling with 2 M sodium hydroxide for 2 hours. The photoinduced alkaline degradation of ACM followed first order kinetics (Fig. 8) which is in agreement with previous reports (3, 30). The rate constant K=0.0276 min^-1^, and t_½_ (0.693/K) was found to be 25 minutes. In acid medium, however, the drug was found to be stable, and this is in agreement with reports which state that the drug has a maximum stability at pH 4 (3, 30).

Upon exposure of a methanolic solution of ACM to Deuterium lamp with λ 254 nm for different time intervals (up to 180 minutes), it was found that only 30% of the drug has been decomposed. The degradation product showed no interference with the assay of ACM as revealed by the high accuracy and precision of the proposed method. The results are abridged in Table 5.

**Table 5:** Application of the proposed method for the determination of acetazolamide in the presence of its degradation product

Parameter	Amount taken of degradation product (μg/ml)	Amount taken of ACM (μg/ml)	Amount found of ACM (μg/ml)	% Found of ACM

	10 μg/ml	10	10.03	100.30;
		20	20.14	100.70
		30	29.94	99.80
		40	39.89	99.73
		50	50.02	100.04
X ± SD				100.11 ± 0.39
Student′s t test				0.93 (2.78)
Variance ratio F test				1.22 (6.94)
	20 μg/ml	10	10.14	101.40
		20	20.06	100.30
		30	29.93	99.77
		40	39.94	99.85
		50	50.07	100.14
X ± SD				100.29 ± 0.66
Student’s t test				0.53 (2.78)
Variance ratio F test				2.36 (6.94)
	30 μg/ml	10	10.15	101.50
		20	20.15	100.75
		30	30.25	100.83
		40	40.09	100.23
		50	49.96	99.92
X ± SD				100.65 ± 0.61
Student’s t test				0.99 (2.78)
Variance ratio F test				2.01 (6.94)

Figures between parentheses are the tabulated t and F values, respectively, at *p*=0.05 (42).

### Mechanism of the reaction

**Figure 9:**
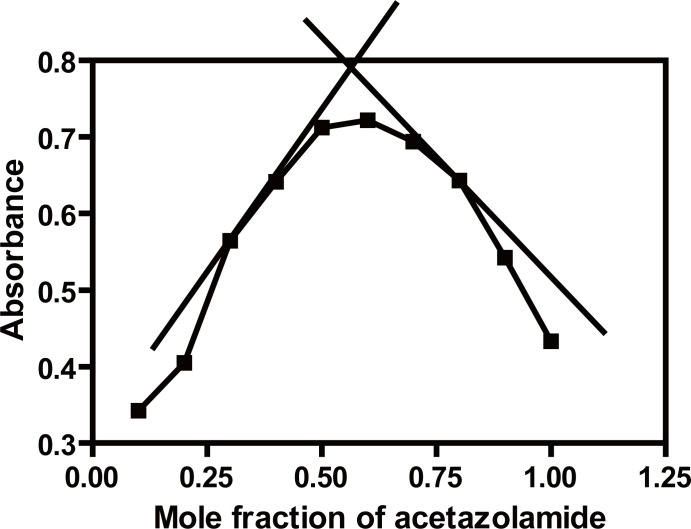
Continuous variation graph for acetazolamide and Pd (II) (5.0 × 10^-3^ M for each of ACM and Pd (II)).

**Figure 10:**
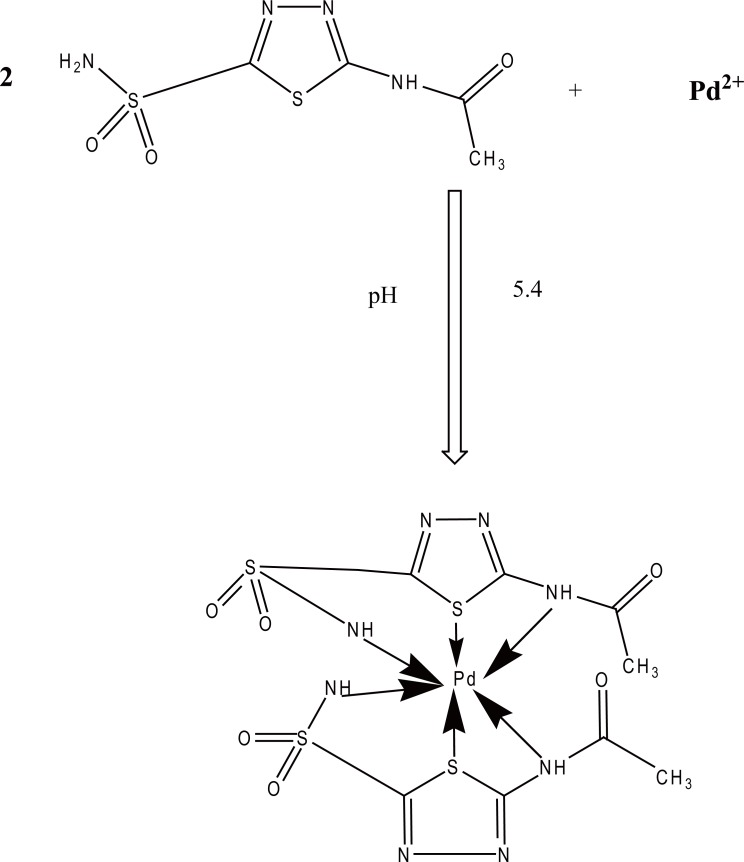
Proposal of the reaction pathway between ACM and Pd (II).

The stoichiometry of the reaction between ACM and Pd (II) has been determined spectrophotometrically by applying Job’s continuous variation method (44), the plot (Fig. 9) reached a maximum value at a mole fraction of 0.6 which indicated the formation of a 2:1 ACM-Pd(II) complex; pointing out that two molecules of the drug react with one molecule of Pd (II). The drug reacts via its sulfur and nitrogen atoms with Pd (II) ion. Based on the obtained molar reactivity and by analogy to a previous study (38), the reaction pathway is proposed to proceed as shown in Fig. 10.

## CONCLUSION

It is found that ACM and Pd (II) can form a yellow coloured complex. A simple and sensitive method was developed for the determination of acetazolamide in pure form and in its tablet. It can measure as low as 2.96 μg/ml with good accuracy. The complex formed did not require a prior extraction procedure. The proposed method could be used for routine quality control and it has some distinct advantages over other existing methods regarding sensitivity, time saving, and a lower detection limit (LOD) of 0.98 μg/ml. It is considered as a stability indicating one.
